# An anthropogenic landscape reduces the influence of climate conditions and moonlight on carnivore activity

**DOI:** 10.1007/s00265-023-03331-9

**Published:** 2023-05-11

**Authors:** Anna Wereszczuk, Andrzej Zalewski

**Affiliations:** Mammal Research Institute, Polish Academy of Sciences, Stoczek 1, 17-230 Białowieża, Poland

**Keywords:** Activity behaviour, Ambient temperature, Snow cover, Carnivores, Moonlight

## Abstract

**Abstract:**

Abiotic factors are limitations that can affect animal activity and distribution, whether directly or indirectly. The objective of this study was to evaluate how abiotic factors influence the activity of two mustelid species inhabiting the same region but different habitats in NE Poland—pine marten inhabits forests and stone marten occupy built-up areas. From 1991 to 2016, we obtained 23,639 year-round observations of 15 pine martens and 8524 observations of 47 stone martens. We explore the influence of ambient temperature, snow cover depth and moonlight reaching the ground and interactions between these variables on the probability of martens’ activity. The activity of pine martens living in natural habitats is more affected by climate conditions and moonlight than that of stone martens living in anthropogenic areas. Pine martens inhabiting forests increased activity when the ambient temperature was above 0 °C and snow cover was absent, as well as when the ambient temperature dropped to − 15 °C and snow cover depth was about 10 cm. Stone marten occupying anthropogenic areas did not reduce their activity if the temperature dropped. Variation of activity in relation to ambient conditions is probably related to pine martens’ behavioural thermoregulation. The pine marten was active more frequently on bright nights, while moonlight intensity did not affect the activity of the stone marten. Our study concludes that complex interactions among abiotic factors concerning different habitats play a synergetic role in shaping carnivore activity and suggest that climate warming may affect the behaviour of both martens.

**Significance statement:**

The survival and reproduction of animals depends on their activity, which is subject to various constraints. We studied the influence of climate conditions and moonlight intensity on the ground on the activity of pine and stone marten. We found that pine martens in natural habitat were greatly impacted by ambient conditions, whereas stone martens in built-up areas were less so. Natural habitats involve limitations related to harsh winters but may mitigate the effects of high temperatures. In contrast, animals living in built-up areas are exposed to higher temperatures in summer, which is of particular importance in the face of climate change. Our results show that the combination of several environmental factors affects animal behaviour and these factors have varying effects in various habitats.

**Supplementary Information:**

The online version contains supplementary material available at 10.1007/s00265-023-03331-9.

## Introduction

The efficient procurement of food is crucial for the survival and reproduction of animals. Therefore, factors affecting animal foraging and activity act as a primary question for ecological research. The activity of animals mainly depends on food availability, thermal conservation (e.g. thermoregulation, including heat loss; Rocha et al. 2022), and intra- or inter-species interactions (e.g. predation risk and/or competition; Halle 2000). Abiotic conditions directly or indirectly influence some of these factors, such as thermoregulation or predation risk (Bonebrake et al. 2020; Lewis et al, 2017; Prugh and Golden 2014). Outside of its thermoneutral zone, an animal will modify the time of activity (Lopes and Bicca-Marques 2017; Terrien et al. 2011; Woodroffe et al. 2017), e.g. by reducing it at high ambient temperature (Mcnutt et al. 2019; Woodroffe et al. 2017) and at very low ambient temperature alike (Zalewski 2000). Behavioural thermoregulation to prevent heat loss or overheating is a ubiquitous animal response. However, ambient temperature is not the only factor affecting activity, and its influence often interacts synergistically with other factors (e.g. snow cover or light). Snow cover reduces the availability of food (Bryce et al. 2022), which should increase activity time even if the temperature is low. At the same time, movement through deep snow increases locomotive costs and metabolic rate (Martin et al. 2020). Moonlight, by increasing predation risk and suppressing prey activity (Gordigiani et al. 2022; Pratas-Santiago et al. 2017; Prugh and Golden 2014), may modify success in acquiring food, regardless of weather conditions. Therefore, an understanding of factors affecting activity should include various abiotic factors and interactions among them.

The simultaneous effect of abiotic conditions on animal activity may be additionally modified in habitats altered by humans. Urban environments create local temperature increases and relative humidity decreases, generated by heat storage in buildings and sealed roads, in contrast to neighbouring nonurban areas (Arya 2001). The urban heat island effect also appears along the urban-rural gradient and occurs to a lower extent in villages, especially as an effect of home heating during winter (Hinkel and Nelson 2007). Higher ambient temperature in an anthropogenic environment mitigates the influence of extremely cold winter temperatures and may extend activity periods of animals vulnerable/exposed to low temperatures (Herrera and Cove 2020). On the other hand, it may accelerate overheating in extremely warm summer temperatures. Faster snow cover melt and snow removal from streets, park paths, and backyards in urban and rural areas potentially reduce the impact of snow cover on animals’ movement and activity. Furthermore, shelters in buildings and anthropogenic food sources enable partial independence from climate conditions in anthropogenic habitats. A disadvantage of inhabiting an anthropogenic landscape is the need for animals to adjust their activity to avoid human encounters, becoming active primarily at night and narrowing the timespan of their potential activity (Mori et al. 2020). Moreover, light pollution may alter the behaviour and interactions of both predator and prey causing some areas and times of day to have too much artificial light to be exploited by predators (Ditmer et al. 2021). Thus, human influence in anthropogenic habitats could potentially be an overriding factor modifying an animal’s activity pattern and/or may relax the impact of abiotic conditions on animal activity (Sogliani et al. 2021).

Weasel-like Mustelids with high basal metabolic rate and energy demands (Casey and Casey 1979; Moors 1977) are more ‘starvation driven’ and need to exploit every opportunity to obtain food; thus, they tend to be active even under unfavourable ambient conditions, even though they are at greater risk of heat and energy loss. The success of hunting overnight, especially in low temperatures and high snow cover depth, which increase energy expenditure, may determine the survival of the individual. Therefore, abiotic factors are of particular importance for the activity of Mustelids. Their activity can be additionally related to prey activity (mainly rodents; Lode 1995), which is determined by the same abiotic factors. However, abiotic conditions may affect the activity of prey in the opposite direction to predators—moonlight intensity suppresses rodents’ activity (Prugh and Golden 2014) due to more optimal hunting conditions and intensified predator activity (Broekhuis et al. 2014). Snow cover increases the survival of rodents because they can move under the snow and are less accessible to some predators (Pucek et al. 1993). The indirect influence of abiotic conditions may modify the activity of small predators also due to avoidance of encounters with dominant predators (Cozzi et al. 2012).

Pine marten (*Martes martes*) and stone marten (*Martes foina*) are closely related Mustelids, with similar body sizes (Wereszczuk et al. 2021). Marten species are characterised by elongated, thin bodies which have significantly more surface area relative to other mammals of the same weight, which determines higher sensitivity to heat loss (Brown and Lasiewski 1972). Both martens exhibit great adaptive plasticity and inhabit a wide range of habitats in most of Europe (Croitor and Brugal 2010; Virgos et al. 2012). However, in contrast to stone martens, pine martens predominantly avoid urban and high human disturbance areas on the spatial scale (Balestrieri et al. 2019; Mergey et al. 2011; Mori et al. 2022). In north-eastern Poland, both species occupy almost separate habitat niches—pine martens occur in deciduous, coniferous and mixed forests, while stone martens inhabit developed areas and human settlements (Wereszczuk and Zalewski 2015). Thus, although they occur in areas with the same abiotic conditions, the influence of these conditions on activity could be different due to, for example, utilisation of different thermal insulation resting sites (e.g. tree hollow by pine marten vs heated building by stone marten) or hunting sites (e.g. forest for pine marten vs barn for stone marten). The activity pattern of stone marten may be partially released from the pressure of abiotic conditions thanks to the colonisation of anthropogenic habitats and thus the magnitude of its activity changes in response to abiotic conditions will be smaller in comparison to pine marten.

Both species are predominantly nocturnal or crepuscular with the potential for more frequent activity during the day in pine marten (Zalewski 2000; Zielinski et al. 1983). While pine martens were active more often in daylight hours, especially in spring and summer, stone martens appear to be strictly nocturnal to avoid human encounters (Gaynor et al. 2018; Wereszczuk and Zalewski 2015). Previous studies have suggested an abundance of prey and avoidance of diurnal predators as the main factors driving activity patterns of martens (Roy et al. 2019; Zielinski et al. 1983) but have also indicated a linear reduction of activity in response to winter thermal stress (behavioural thermoregulation; Zalewski 2000). However, studies that consider the influence of various abiotic factors on marten activity are scarce. In this study, we tested whether various abiotic conditions, including temperature, snow cover depth, and moonlight and two-way interactions among these variables, affected the probability of pine and stone marten activity within the same region but different habitats. We expected the impact of abiotic conditions would differ depending on the natural and anthropogenic habitats occupied by those two similar species. We predicted that the activity of both species would decrease with (1a) decreasing ambient temperature, (1b) increasing snow cover, and (1c) decreasing moonlight intensity, or alternatively, (2a) activity of pine marten would decrease with decreasing ambient temperature, increasing snow cover, and decreasing moonlight intensity but (2b) stone marten would be unaffected by the abiotic conditions.

## Materials and method

### Study area

**Fig. 1:**
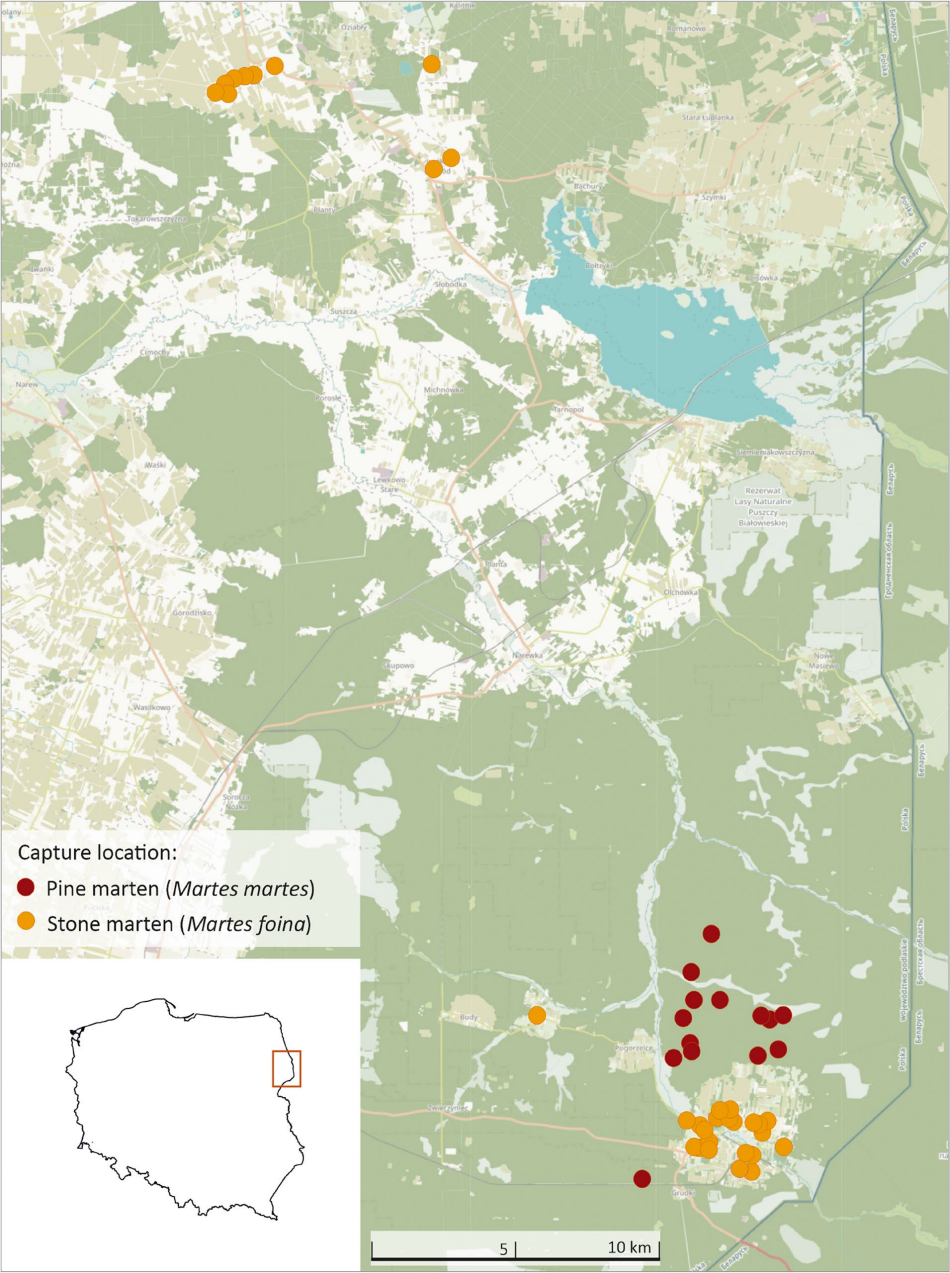
Study area and locations of captured pine martens and stone martens from 1991 to 2016 in NE Poland

The study was conducted in north-eastern Poland (Fig. 1). The region is characterised by extensive agriculture, low human population density (27–49 people/km^2^), and a moderate rate of urbanisation (38–50%; Polska w liczbach 2020). Martens were captured in Białowieża Primeval Forest (BPF; E 23.8460, N 52.7457) covering 1250 km^2^ in three villages inside the BPF and three villages in a mosaic of agricultural and forest patches 10–15 km to the north from BPF. BPF is one of the largest forest complexes in Europe and one of its last nonaltered habitats (Samojlik et al. 2013). In the region, pine martens occupied predominantly deciduous forests and forest patches, while stone martens inhabited almost only developed areas (Wereszczuk and Zalewski 2015). Therefore, most of the pine martens were captured in Białowieża National Park (Zalewski and Jędrzejewski 2006)—an old-growth forest composed of oak *Quercus robur*, hornbeam *Carpinus betulus*, spruce *Picea abies*, lime *Tilia cordata,* black alder *Alnus glutinosa*, ash *Fraxinus excelsior*, pine *Pinus silvestris*, maple *Acer platanoides*, and several other tree species. The stone martens were captured in villages mainly located in the centre of BPF, and the distance between sites of pine and stone marten capture was approximately 3 km.

The climate of the study area is transitional between Atlantic and continental types. During the study period (1991–2016) the mean annual ambient temperature was 7.3 °C and the mean annual precipitation was 636.3 mm. The minimum temperature occurred in January (monthly average − 3.3 °C; daily minimum temperature in the range from − 34.6 to 7.1 °C) and the maximum in July (monthly average 18.7 °C; daily maximum temperature in the range from 12 to 34.6 °C). The mean annual number of days with snow cover was 76 and varied between 132 days in 1996 and 15 days in 2015. A high variation in ambient temperature and snow cover allowed us to analyse the influence of climate variation on marten activity. The influence of artificial light was not analysed in this study due to it was turned off every day after midnight in villages in this region, i.e. in the times of the highest stone marten activity (Wereszczuk and Zalewski 2015).

Map was created using the R package ‘tmap’ (Tennekes 2018) with the ‘Humanitarian’ map style of OpenStreetMap (https://github.com/hotosm/HDM-CartoCSS; accessed on 27 March 2023).

### Trapping and radio tracking

Between April 1991 and September 2014, 15 pine martens (8 females and 7 males), and between May 2011 and March 2016, 47 stone martens (24 females and 23 males) were captured in live traps and collared. Captured martens were anaesthetised using 15 mg/kg ketamine and sexed, weighed and measured to determine body length. Martens were fitted with radio collars (AVM, Lotek, or ATS) that weighed 12–25 g and were less than 2% of the weight of the individual. The life span of the transmitter was 5–12 months. After recovery, martens were released at the site of capture. More information about trapping is provided in Wereszczuk and Zalewski (2019) and Zalewski and Jędrzejewski (2006).

We tracked collared martens using a receiver (Telonics or Yaesu, FT 817 ND) and H-shaped or 3-element Yagi antenna. The radio-collared martens were located by an observer on foot one or two times per day (once in daylight and/or night) at least four times per week. Determination of the marten's activity status (active/inactive) took 5–15 min, during which activity was determined 2–3 times. Bearings were obtained from a distance < 500 m, usually about 300 m in the forest and 200 m in the village. Besides obtaining one location per day, some individuals were monitored continuously for 4–24 h sessions, and observations were recorded at 15-min intervals to assess marten activity. Martens were characterised as active when the signal frequently switched pulse amplitude, or inactive when the signal pulse amplitude remained constant. It was not possible to record data blind because our study involved focal animals in the field.

### Climate variables and moonlight illumination

To analyse the influence of climate conditions on martens’ activity, we obtained hourly air temperature and daily snow cover depth from the nearest climatic station of the Polish Institute of Meteorology and Water Management, National Research Institute (E 23.8659, N 52.6999). Meteorological data were obtained using the R package ‘climate’ (Czarnecki et al. 2020).

In most studies, the influence of moonlight on mammals’ activity is analysed by the lunar cycle (Cozzi et al. 2012; Peeva et al. 2022; Prugh and Golden 2014). This approach, however, does not take the amount of on-the-ground illumination throughout each night into account (Śmielak 2023). In our study, we used a new approach that combines the position of the moon in the sky, the brightness of the moon’s face and several physical properties of light propagation to count predicted moonlight illumination, relative to an ‘average’ full moon, for a given place and time. Moonlight illumination was characterised using the package ‘moonlit’ to obtain the ambient intensity of moonlight on the ground (Śmielak 2023) and for latitude 23° E intensity of moonlight varied from 0 to 0.6. Extinction coefficient *e* (magnitude per air mass) was set at 0.24, corresponding to 500 m asl. For each day, the time of sunset and sunrise was obtained from the above package to define night, understood as the time between 2 h after sunset and 2 h before sunrise, which corresponded to the period without the influence of sunlight.

### Statistical analysis

We analysed the probability of marten activity in response to climate variables and moonlight illumination with a generalised additive model (GAM; package ‘mgcv’; Wood 2020) using activity as the binomial response variable (nonactive, active). For each species, we analysed two separate models: (1) probability of daily activity (both daylight and nighttime) and (2) probability of activity only during hours of darkness. We performed two models to analyse the influence of ambient conditions for general marten activity and the period of the highest activity only. Analysing daily activity, we used the sex of the marten, the ambient temperature, the logarithm of snow cover depth, and the time of the day as explanatory variables. In addition, the time of day and temperature as well as the time of day and snow cover depth were fitted as two-way interactions. Next, we included moonlight illumination along with ambient temperature, snow cover depth and sex in the model of night activity, and we fitted temperature and snow cover depth as well as temperature and moonlight as two-way interactions. Variance inflation factors (VIFs) indicated noncollinearity among all abiotic explanatory variables (VIFs < 2) and thus were included in the models together. In both type of models, explanatory variables, except sex, were fitted with splines. Cyclic cubic regression splines were used for smoothing of time of day (in the diurnal model), as recommended for cyclic variables (Wood 2017). As the same individual was recorded multiple times, we set the individual’s ID as a categorical random factor in both models. We assume the same level of curvilinearity in the effect of explanatory variables (time of day, temperature and snow cover depth in daily models; temperature, snow cover depth and moonlight in nocturnal models) for both species; thus, we set the same smoothing ranges for each variable. Models were specified using binomial distribution, an identity link function, and a REML approach. We used ‘vis.gam’ function to produce contour plots of model predictions (‘mgcv’ package). Predicted effects of abiotic variables from the models were restricted to avoid undue extrapolation, and thus were controlled within the range of the original covariate values using too.far = 0.20. All statistical analyses were performed in R 4.0.3 (R Core Team 2020).

## Results

### Daily activity

**Fig. 2:**
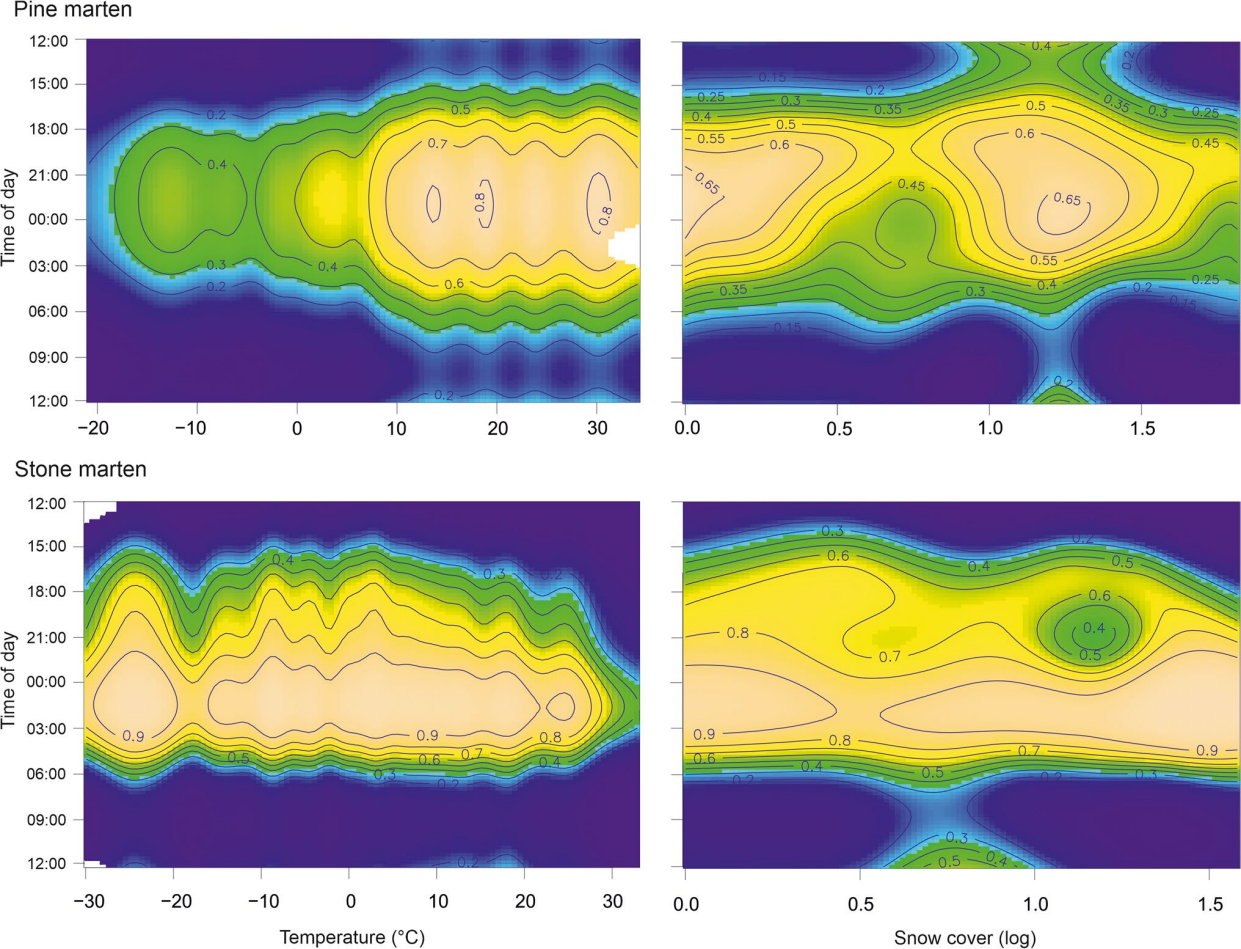
Predicted effect of temperature, daily snow cover depth, and time of day on the probability of daily activity of pine marten and stone marten. The logarithm of snow 0.5, 1 and 1.5 corresponds to 3 cm, 10 cm and 31 cm of snow cover depth, respectively

**Fig. 3:**
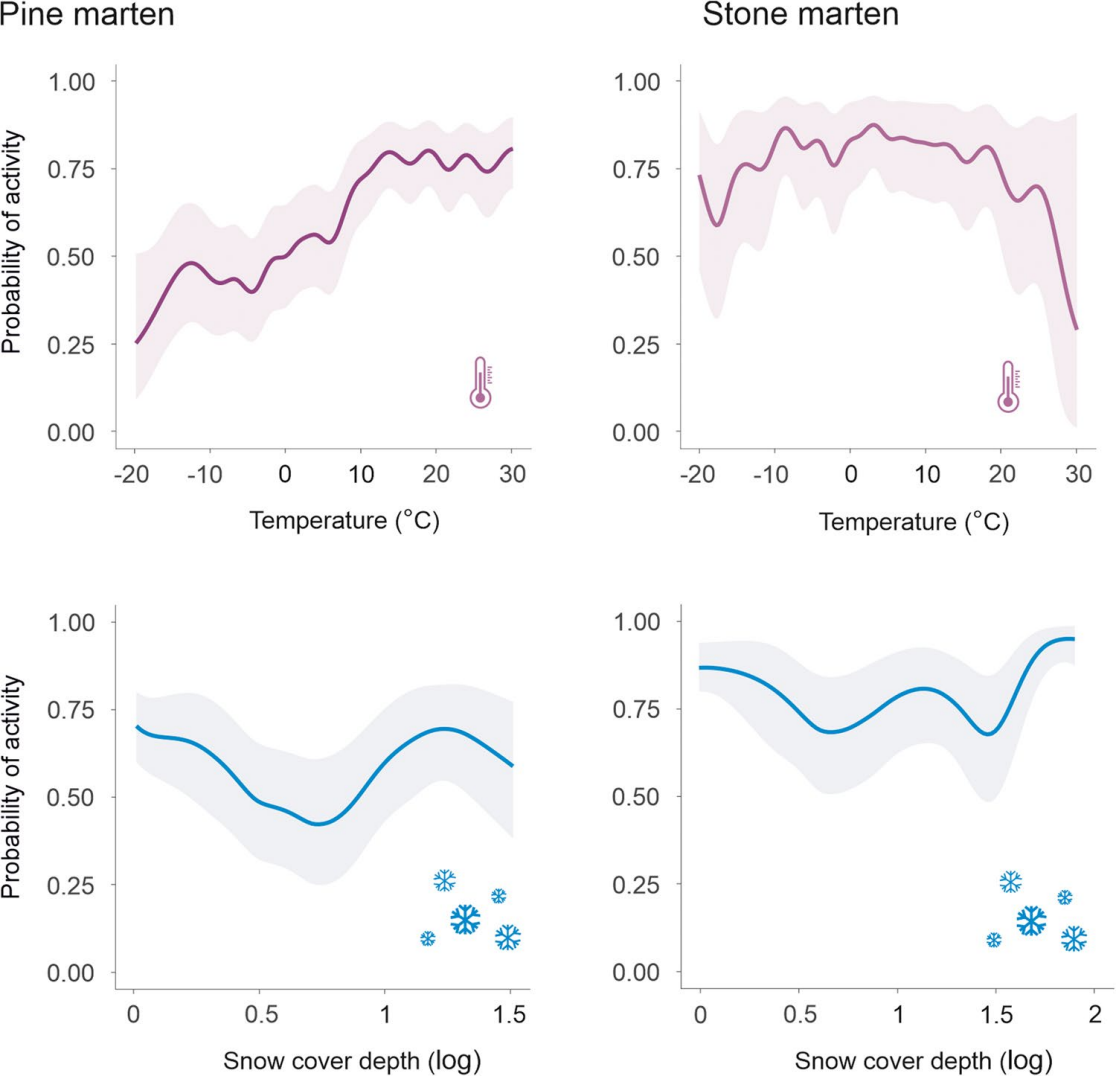
Predicted probability of daily activity (with 95% confidence interval) of pine marten and stone marten in relation to ambient temperature and snow cover depth (logarithmic). Predictions are shown for midnight and males. Snow cover log of 0.5, 1 and 1.5 corresponds to 3 cm, 10 cm and 31 cm of snow cover depth, respectively

We obtained 23,639 observations of 15 collared pine martens (resulting in a total of 5910 h of monitoring) and 8524 of 47 collared stone martens (resulting in a total of 2131 h of monitoring). The daily activity of pine and stone marten was strongly associated with the time of the day and its interaction with temperature or snow cover depth. Pine martens were active from 15:00 to 7:00, and the probability of activity was at a stable level (about 0.75–0.80) at a temperature between 10 and 30 °C and decreased almost linearly with decreasing ambient temperature down to the very low probability of activity level (0.25) at ambient temperature − 30 °C. The probability of pine marten activity was lowest when snow cover depth was below 3 cm and increased at both deeper and shallow snow cover. The activity of stone marten was related more to time of day than temperature or snow cover. Stone martens were active from 16:00 to 6:00, reducing activity only when the ambient temperature was above 25 °C, and snow cover depth had only a slight effect on the probability of their activity (Table 1, Figs. 2 and 3).

**Table 1:** Results of generalised additive models of interactive effects of time, temperature or moonlight, and sex on stone and pine marten activity. Individuals’ IDs were added as a random effect

Variable	Pine marten			Stone marten		
	Estimate ± SE or edf	Test statistics	*p*	Estimate ± SE or edf	Test statistics	*p*
Daily GAMs						
Parametric terms		z			z	
Intercept	− 1.63 ± 0.19	− 8.48	< 0.001	− 1.35 ± 0.18	− 7.62	< 0.001
Sex (m)	0.43 ± 0.28	1.55	0.12	0.43 ± 0.25	1.73	0.08
Smooth terms		*X*^2^			*X*^2^	
Time of day	3.91	156.49	< 0.001	3.67	158.32	< 0.001
Temperature	1.00	1.84	0.17	1.00	0.09	0.77
Snow cover	1.00	1.89	0.17	1.00	2.62	0.10
Time × temperature	20.12	251.21	< 0.001	23.10	149.89	< 0.001
Time × snow cover	23.84	251.35	< 0.001	21.19	164.38	< 0.001
Nocturnal GAMs						
Parametric terms		z			z	
Intercept	− 0.40 ± 0.21	− 1.89	< 0.001	0.83 ± 0.25	3.39	< 0.001
Sex (m)	0.87 ± 0.30	2.93	0.003	0.49 ± 0.35	1.40	0.16
Smooth terms		*X*^2^			*X*^2^	
Time of day	6.26	43.59	< 0.001	7.60	87.78	< 0.001
Moonlight	2.83	9.88	0.03	1.00	1.42	0.23
Temperature	7.05	178.79	< 0.001	1.00	25.73	< 0.001
Snow cover	3.49	31.10	< 0.001	1.12	8.26	0.01
Temperature × moonlight	0.0001	0	0.03	0.71	155.81	0.10
Temperature × snow cover	0.002	0.001	0.002	0.000035	0	0.35

### Nocturnal activity

**Fig. 4:**
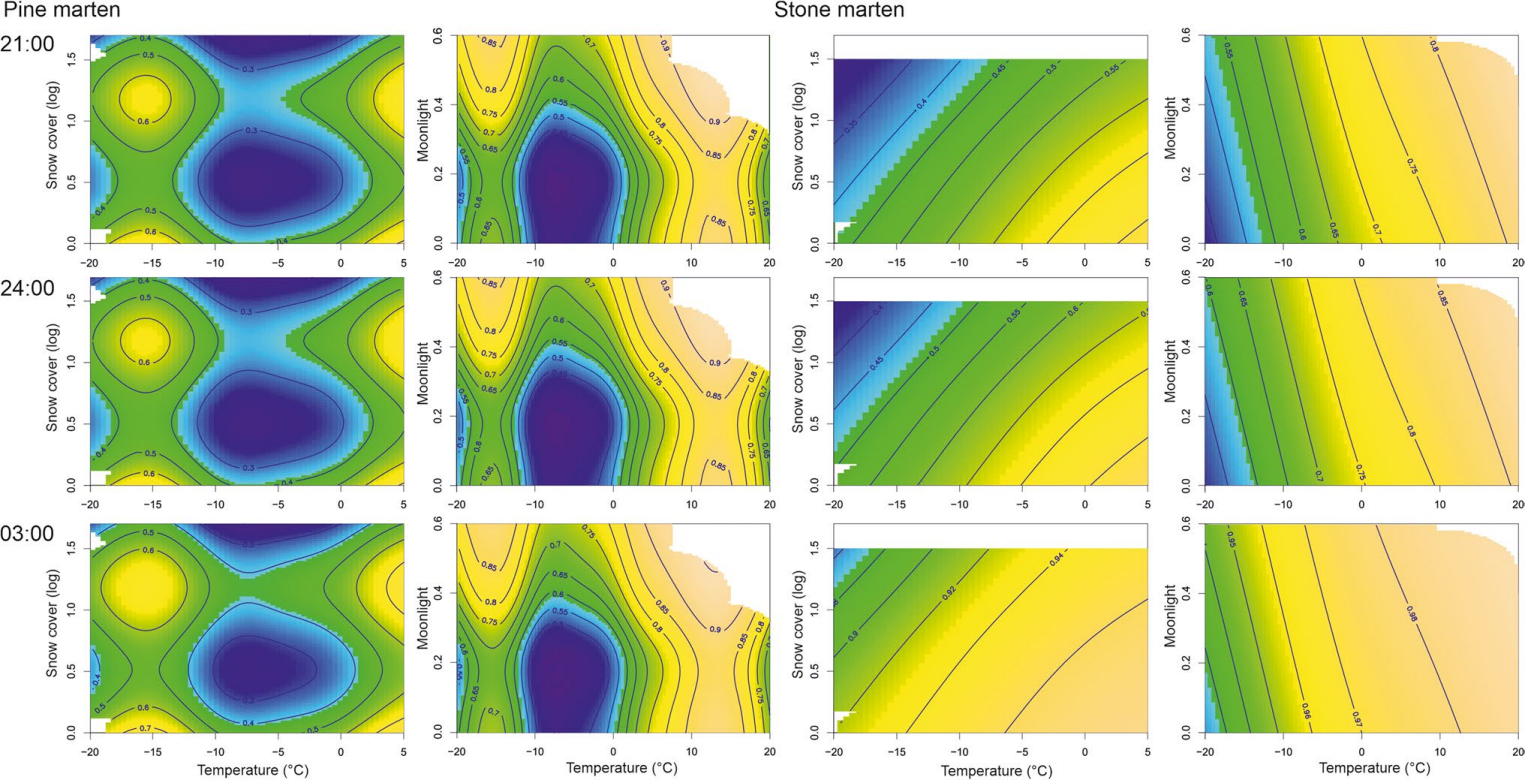
Predicted effect of temperature, daily snow cover depth, and intensity of moonlight on the ground on the probability of nocturnal activity of pine marten (*Martes martes*) and stone marten (*Martes foina*) at 21:00, 24:00, and 3:00 (UTC+02:00). The logarithm of snow 0.5, 1 and 1.5 corresponds to 3 cm, 10 cm and 31 cm of snow cover depth, respectively

The probability of pine marten activity at night was the highest when the ambient temperature was above 0 °C, slightly decreased when the ambient temperature exceeds 15 °C, and strongly decreased when the ambient temperature fell to − 10 °C. With a further decrease in ambient temperature (− 15 °C), the probability of pine marten activity increased, followed by a decrease together with a further decrease in temperature (− 20 °C). The probability of pine marten activity at night was the highest at 0 cm or 10–30 cm snow cover depth and when moonlight was 0.6. Nocturnal activity of stone marten was positively correlated with temperature and negatively correlated with snow cover depth without significant interaction of those variables. Moonlight did not influence the nocturnal activity of stone marten (Fig. 4).

**Fig. 5:**
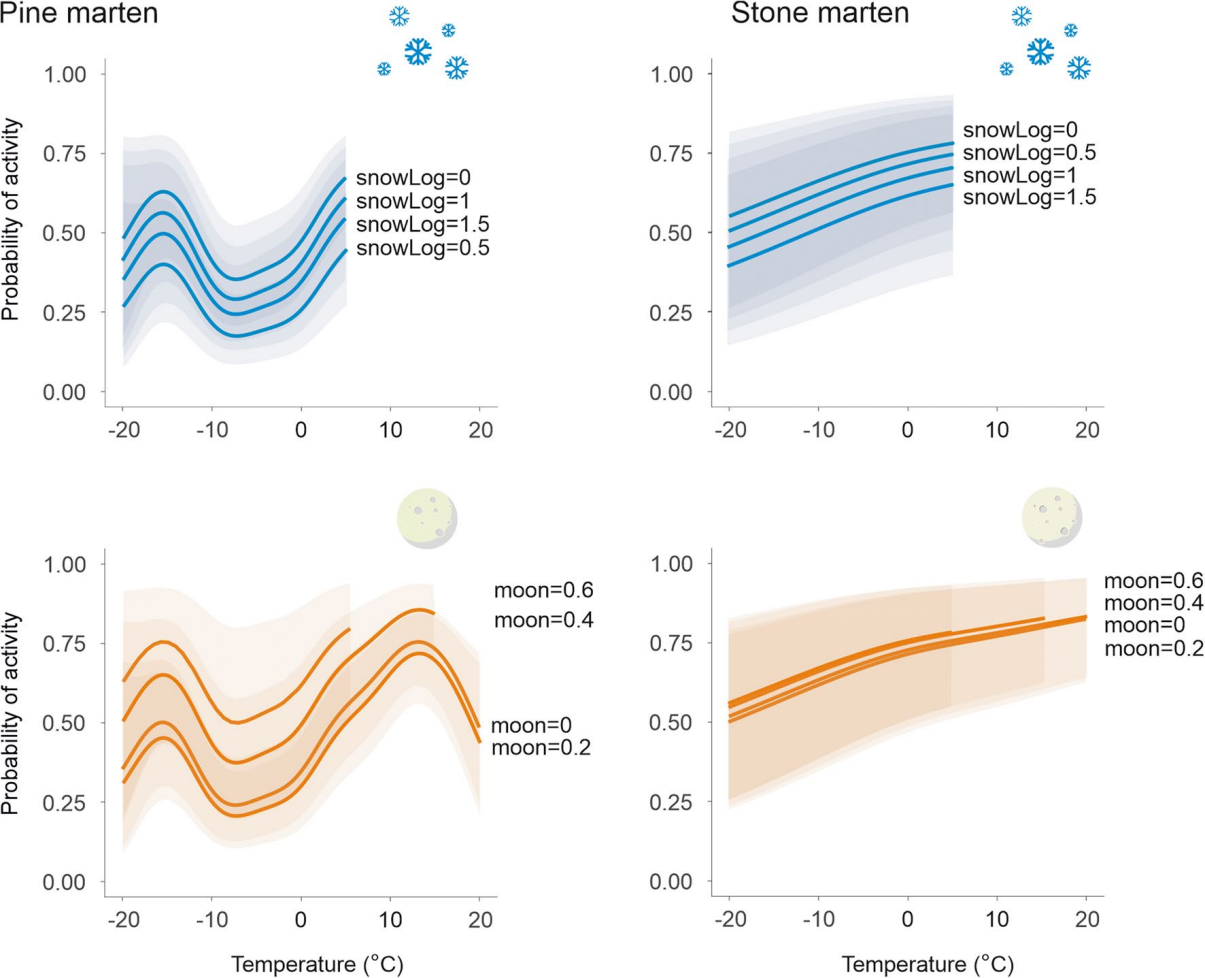
Predicted probability of nocturnal activity (with 95% confidence interval) of pine marten (*Martes martes*) and stone marten (*Martes foina*) for temperature and snow cover depth (logarithmic) and intensity of moonlight on the ground. Predictions are shown for midnight and males. Snow cover log of 0.5, 1 and 1.5 coresponds to 3 cm, 10 cm and 31 cm of snow cover depth, respectively. Predictions of activity at different moonlight levels have been presented up to 5 °C for moonlight of 0.6 and up to 15 °C for moonlight of 0.4, because those moonlight intensities do not reach the Earth's surface at the latitude of 23E in the spring and summer, respectively (see lack of data on Fig. 4)

The probability of pine marten activity at night changed nonlinearly depending on all three analysed ambient factors. The probability of pine marten activity along with temperature changes was the highest in the absence of snow cover and the lowest with 3 cm of snow cover, with middle activity probability at higher values of snow cover depth (10–30 cm). While pine marten activity increased with the intensity of moonlight across temperatures, snow cover depth and moonlight intensity did not influence stone marten activity regardless of ambient temperature (Fig. 5).

## Discussion

Our findings provide evidence that ambient conditions strongly affect the activity of pine marten inhabiting natural habitats, while this constraint is relaxed in stone marten inhabiting anthropogenic areas. For pine marten inhabiting forests, we identified two sets of values of abiotic conditions that increase the probability of nocturnal activity. First, when the ambient temperature was above 0 °C and snow cover was absent; second, when the ambient temperature dropped to − 15 °C and the snow cover depth was about 10 cm. The stone marten occupies anthropogenic areas and is partially independent of ambient conditions. The probability of its activity increased linearly with the rise of temperature and with the drop in snow cover depth, but the variability of activity depending on these climatic factors was much lower than in the pine marten. Additionally, the pine marten was active more frequently on bright nights, when the intensity of moonlight reaching the ground was above 0.2, unlike the stone marten, the activity of which was not at all affected by moonlight intensity.

The findings of this study need to be considered with some limitations. Firstly, we studied pine martens in the natural environment (forest) and stone martens in the anthropogenic environment (village). We did not track stone martens in the natural environment and pine martens in the anthropogenic environment because they do not use these environments in our study area. However, since both marten species have similar body sizes and shapes, we expect that climatic factors will affect them equally and that the habitat used by the species is responsible for the differences in their activity in response to ambient conditions. Secondly, the intensity of moonlight reaching the earth can be modulated by cloud cover, but we were unable to include this parameter in our analyses. It would weaken the observed influence of moonlight on marten activity but since we are comparing its influence between marten species, this would not strongly affect our results. However, the influence of cloud cover should be addressed in future research to further investigate the impact of moonlight on animal activity.

Both martens were primarily nocturnal and crepuscular, in line with previous research (Herr and Roper 2022; Jędrzejewski et al. 1993; Posillico et al. 1995; Zalewski 2000; Zielinski et al. 1983). However, the martens’ daily activities varied in different ways in response to ambient climate conditions. In harsh weather conditions, pine martens, like other Mustelids occupying natural habitats, reduce their mobility and select thermally protective resting microsites to reduce energy expenditure and thermoregulation costs (Joyce et al. 2017; Zalewski 2000; Zub et al. 2009). Weasel-like Mustelids have a very high basal metabolic rate, on average twice as high as that of mammals of similar size (Casey and Casey 1979; Moors 1977), which constitutes up to 70% of their daily energy expenditure (Zub et al. 2009). The cost of thermoregulation increased with decreased ambient temperature (Rezende and Bacigalupe 2015). For example, the energy demands, and thus metabolic rate, of polecats (*Mustela putorius*) increased with the decrease of ambient temperature below 20 °C, and at − 10 °C, their metabolic rate was about twice as high as at + 20 °C (Korhonen et al. 1983). To avoid heat loss caused by exposure to low temperatures, martens reduce their activity. However, reduction in activity over time can be relatively short due to high metabolic rate and small fat reserves. Thus, enhanced activity of pine marten in very low temperature and deep snow may result from the brief depletion (lasting a few days) of energy reserves after a period of reduced activity when weather conditions worsened. In general, high and very low temperatures as well as deep snow also impede the mobility of other carnivores (Bryce et al. 2022; Jędrzejewski et al. 1993) and increase their energy demands.

In contrast to pine marten, stone marten did not reduce their daily activity in relation to the decrease in ambient temperatures. This could be explained partly by the microclimate in the village, but we suggest that this factor affected activity to a lower extent, as the differences between ambient temperatures in both habitats were low. The selection of microsites during rest was probably more important. Stone martens locate resting microsites mostly in buildings (some of them heated in winter), in contrast to pine martens, which mainly use tree cavities and squirrel nests (Herr et al. 2010; Larroque et al. 2015; Zalewski 1997). Built-up areas provide well-insulated microsites with stable ambient temperatures in which stone marten may reduce energy expenditure during resting. In winter, buildings are considered an important resource for other Mustelids that inhabit rural areas (e.g. polecat; Weber 1989). Furthermore, buildings provide an extra, at least partial food source (such as house mice and rats). Why is the stone marten more active than the pine marten during colder days if it uses less energy when resting? First off, stone martens require more food because they are larger than pine martens in this region, at 1.35 vs 1.14 kg on average for both sexes (Wereszczuk et al. 2021; Wereszczuk and Zalewski 2015). Second, in contrast to pine martens, which eat mostly rodents in the winter, stone martens consume a high proportion of fruits, even in winter, as well as food waste (Czernik et al. 2016; Jędrzejewski et al. 1993; Zalewski 2007). Stone martens likely forage for longer to gather enough energy from less energy-rich food. Furthermore, developed areas may facilitate the mobility of stone martens (and reduction of energy expenditure) when there is large snow cover due to patches of snow being removed by humans. Stone martens inhabiting urbanised areas likely have lower costs of mobility, and therefore remain active for longer in harsh weather. The comparison of both martens’ activity may suggest that, like for other Mustelids, the declining activity of pine marten in cold winter temperature is disadvantageous. On colder days, the daily energy expenditure increases, but the marten is active only 25% of the day. Taking into account that martens have small digestive tracts and small fat reserves which limit the amount of food they can consume and the time before fat stores are depleted (Buskirk and Harlow 1989), this decline should be compensated by other mechanisms that enable the conservation of energy.

Due to the large variation in daily activity level, our first model used interactions between time of day and climatic conditions (temperature or snow cover). These analyses did not include the interaction of ambient conditions on marten activity. Therefore, in the following model, we analysed only nocturnal activity (period of the highest marten activity) and interactions between temperature and snow cover or moonlight. Analyses of nocturnal activity indicate that abiotic conditions play a synergetic role in shaping carnivore activity in natural but not urban habitats. Interaction between temperature and snow cover has no significant influence on stone marten activity. In contrast, pine marten activity generally declines when the ambient temperature falls, but the magnitude of the decline is also related to snow cover and moonlight in a nonlinear way. The decrease of activity is greater at temperatures ranging from − 5 to − 10 °C and with a thin layer of snow (around 3 cm). In this condition, rodents are not active under the shallow snow cover, yet they are clearly apparent in the white snow. This could potentially improve the hunting success of pine marten, and may be confirmed by research, which found that the proportion of rodents in the marten diet (primarily bank voles *Myodes glareolus*) is higher in years with shallow snow, and declines when snow cover heightens (Jędrzejewski et al. 1993). With more snow covering the ground, rodents active under the snow are less available for martens, and the pine marten increases its activity. Pine marten activity increases when the ambient temperature drops below − 15 °C, particularly when there is a substantially higher snowpack. In the colder winter months, pine martens search for larger prey, such as squirrels, or feed on ungulate carcasses (Zalewski 2000). The availability of larger prey is potentially lower than smaller prey. These synergistic effects of climatic condition on marten activity are subject to change as climate change progresses, first due to a lack of snow in the study area and rising winter temperatures (Wereszczuk et al. 2023). Considering the influence of different factors interaction placed in the context of the inhabited environment, we may better understand adaptation to ongoing climate changes.

Analysis of the influence of moonlight in interaction with ambient temperature showed moonlight to be an important abiotic factor affecting the nocturnal activity of pine marten but not stone marten. Most of the studies to date have analysed only the effect of moon phases on the activity of animals (e.g. Cozzi et al. 2012; Peeva et al. 2022). We used an alternative approach to obtain moonlight intensity on the ground based on the position of the moon, the brightness of the moon’s face, and several physical properties of light propagation, which is the proper measure of the amount of moonlight of real ecological importance (Śmielak 2023). Martens detect prey visually, thus increased moonlight levels may help them hunt, as is the case for other nocturnal mammals (Prugh and Golden 2014). On the other hand, moonlight illumination suppresses prey activity due to the higher pressure of predators (Pratas-Santiago et al. 2017; Prugh and Golden 2014). Our study shows that pine martens were more active during periods of greater moonlight intensity, which may be related to the need for prolonged activity due to the lower availability of rodents, which reduce their activity under these conditions. However, martens are also more susceptible to predation under these conditions.

In anthropogenic areas, the effect of moonlight may be masked by artificial light, which is much stronger than the light of the full moon (Falchi et al. 2011). However, Willems et al. (2022) indicated that artificial light cannot reduce the foraging activity of rodents as moonlight does. Accordingly, predators may not change their activity pattern in response to artificial light, either. In our study area, artificial light was turned off after midnight, when the probability of stone marten activity was very high (> 0.9), although not affected by moonlight (see Fig. 4). It is also possible that stone marten increases activity in the absence of artificial light, but street lights only occur locally (along main roads) so their impact is probably limited. This may again depend on the food the stone marten eats (fruit and human food waste), the acquisition of which does not require as much light. Additionally, the reduced number of tourists visiting the village due to COVID-19 lockdowns caused a shift in the peak time of stone marten activity to before midnight, even though artificial lighting was left on until that time (Wereszczuk and Zalewski 2022). Thus, it seems that stone martens become strictly nocturnal to avoid human confrontation (Herr and Roper 2022), and, regardless of abiotic conditions, they remain most active between midnight and 6:00, when the probability of human encounter is the lowest.

In general, living in an anthropogenic environment comes with potential benefits, such as stable resource availability, a constant level of food waste or more stable weather conditions; costs include anthropogenic noises, light pollution and human disturbance (Barrueto et al. 2014; Gaynor et al. 2018; Raap et al. 2015; Shannon et al. 2016). The periods of activity among animals inhabiting urban areas are often determined by periods of human inactivity, limiting the time of animal activity to a short part of the night. Synanthropisation released the stone marten from the pressure of harsh climate conditions, which enabled its on-going colonisation of Europe (Wereszczuk et al. 2017). Nowadays, climate change and milder winters may contribute to its further expansion in north-eastern Europe and demographic expansion in central Europe. Thus, rising temperatures and milder winters may benefit animals which have better morphological or behavioural adaptations to warm climates. However, as the climate continues to change, the ‘heat islands’ in urban areas on days with extremely high summertime temperatures make conditions unfavourable for stone martens. When the temperature rises to 25 °C, their activity drops. Additionally, they spend time in cooler micro-sites during the day (such as underground root cellars), which suggests that the typically used micro-sites in stables or attic in homes are too warm. Therefore, further climate change may also negatively affect stone martens that inhabit anthropogenic habitats.

## Conclusions

Abiotic conditions directly affect animals’ thermoregulation and food acquisition, and thus their activity. The type of environment they inhabit modifies the influence of abiotic factors. Although it involves a number of limitations, occurrence in anthropogenic environment mitigates the impact of harsh winters. On the one hand, animals living in built-up areas are exposed to higher temperatures in summer, which is of particular importance in the face of climate change. On the other hand, occurrence in natural habitats involves limitations related to harsh winters but may mitigate the effects of high temperatures. The complex interactions among abiotic factors in association with different habitats play a key role in understanding animal activity patterns, their distribution and possibilities of future expansion. This will be particularly important for understanding populations’ threats in the context of climate change.

## Supplementary information

ESM 1

## Data Availability

All data generated and analysed during this study are included in this published article as a supplementary file (ESM 1).
